# Fruit and vegetable consumption among adults in Namibia: analysis of a nationally representative population

**DOI:** 10.15171/hpp.2018.40

**Published:** 2018-10-27

**Authors:** Sanni Yaya, Ghose Bishwajit

**Affiliations:** ^1^Faculty of Social Sciences, School of International Development and Global Studies, University of Ottawa, Ottawa, Canada; ^2^Institute of Nutrition and Food Science, University of Dhaka, Dhaka, Bangladesh

**Keywords:** Demographic and Health Survey, Fruit and vegetable consumption, Socio-demographic factors, Namibia

## Abstract

**Background:** Prevalence of F&V consumption in Namibia is not known. In this study we aimed to address this gap by using nationally representative data with the objectives of measuring the prevalence of adequate F&V consumption among adult men and women and their socio demographic determinants.

**Methods:** This study is based on data from Namibia Demographic and Health Survey (NDHS2013). Sample population were 14 185 men and women aged between 15 and 49 years.Amount of fruit and vegetable consumption was measured by self-reported frequencies and was defined as adequate (at least 5 servings/day) according to World Health Organization (WHO)guidelines.

**Results:** Overall, only 4.3% (3.8-4.9%) of the men and women reported consuming at least 5 servings of F&V a day, with the percentage being slightly higher among women (4.8%,95% CI=3.7-6.2) compared with men (4.2%, 95% CI=3.6-4.8). In the multivariable analysis,education level and household wealth status appeared to be the only factors associated with adequate F&V intake. Men and women who had primary level education had higher odds of eating at least 5 servings of F&V a day compared with those who had no education. Regarding wealth status, men and women from non-poor households had respectively 2.13 times(OR=2.13, 95% CI=1.01-4.48) and 2.2 times (OR=2.19, 95% CI=1.56-3.38) higher odds of eating at least 5 servings of F&V a day.

**Conclusion:** Only a small proportion of the men and women consumed adequate amount of F&V on daily basis. Having primary level education and non-poor household wealth status were positively associated with adequate amount of F&V intake.

## Introduction

Low intake of F&V is recognised as a leading dietary risk factor contributing to global burden of disease. A growing volume of studies suggest that F&V consumption is associated with better subjective health, psychosocial well-being, greater longevity, and significantly lower burden of all-cause mortality.^1-4^ Some of the notable beneficial outcomes include reduced risk of chronic diseases including diabetes, cardiovascular diseases (CVDs), several types of cancer (e.g. breast and lung cancer). Healthy people who consume cruciferous vegetables was also reported to lower likelihood of developing intestinal, bowel, thyroid, pancreatic and lung cancer. Recent investigations are claiming that F&V can help maintain healthy body weight and weight loss management. The exact mechanism is not yet completely understood, however it is believed that various anti-adipogenic bioactive molecules that can regulate the formation of mature adipocytes are the responsible for the curbing effect on obesity.^5-8^ It is believed that several phytochemicals (e.g. epigallocatechin-3-gallate, genistein, and resveratrol) function as potent anti-obesity agents via their role in suppression of adipogenesis and lipid accumulation, inducing lipolysis and apoptosis of existing adipocytes, thereby reducing the amount of adipose tissue.^8,9^ Adiposity is closely related to biomarkers of oxidative stress and inflammation and a diet rich in F&V can modify the adiposity related metabolic biomarkers in overweight women. F&V rich in calcium and magnesium content play vital role in bone development in children, maintaining bone health and help prevent osteoporosis among adults.^10-12^

Apart from physical health and well-being, F&V consumption is also reported to have potential role on promoting psychological health and preventing psychological morbidity especially among the adult and elderly.^13-15^ Accumulating evidence in the field of nutritional psychology maintains that changing dietary behavior (e.g. shifting to highly processed food) has greatly contributed to the rising rates of depression and anxiety among both men and women.^16-18^ Although pharmacological treatments have been the mainstay of therapeutic approach for depression and anxiety symptoms disorders, non-pharmacological aspects (e.g. dietary management) of psychological disorders have also been gaining traction among researchers and practitioners. As such, there has been a growing interest in improving F&V consumption as a dietary strategy of preventing non-communicable chronic diseases (NCDs) as well as mental illnesses.

Despite the fact that the prevalence of NCDs such as obesity, CVDs, type-2 diabetes is expanding at an alarming rate in low- and middle-income countries (LMICs), healthcare systems in the LMICs have so far been burden with malnutrition and infectious diseases, with relatively little research attention and funding allocation for epidemiological studies in the fields of lifestyle medicine and chronic diseases.^19-21^ To date, only a handful of studies have been conducted on the F&V consumption behavior in an African setting. Dietary behavior is a multifactorial construct that is influenced by various individual, demographic, environmental and sociocultural determinants. These factors are expected to vary across and within population, and consequently may influence people’s dietary behavior in diverse ways and magnitudes. With this in mind, we undertook the present study to generate evidence of F&V consumption and its determinants in Namibia, one of the fastest growing economy in located in the Southern Africa. Socioeconomic progress in the past decade has been accompanied by transformation in the diseases and demographic as well as lifestyle behavior of the population.

## Materials and Methods

### Participants and procedures

The 2013 Namibia Demographic and Health Survey (NDHS) was the fourth to be conducted in the country as part of the global DHS programme. NDHS serves as a periodic update on a wide range of demographic and health indicators. The survey was implemented by the Ministry of Health and Social Services (MoHSS) in collaboration with the Namibia Statistics Agency (NSA) and the National Institute of Pathology (NIP) with technical assistance by ICF (Inner City Fund) International and financial support from the Government of Namibia, the United States Agency for International Development (USAID), and the Global Fund. For sampling, a two-stage stratified technique was employed. In the first stage, 554 enumeration areas (primary sampling units) were randomly selected from urban (269) and rural areas (285). In the second stage, a fixed number of households (20) were selected from the each of the urban and rural clusters. From the selected households, 9940 women and 5271 men were identified as eligible for interview, of whom final interview was completed by 10 018 women (response rate = 92%) and 4481 men (response rate = 85%). Field work for the survey lasted from May to September of 2013. More information regarding the survey are available in the DHS report: The Nambia Ministry of Health and Social Services (MoHSS) and ICF International. 2014. The Namibia Demographic and Health Survey 2013. Windhoek, Namibia, and Rockville, Maryland, USA: MoHSS and ICF International.

### Measures

The outcome variable was adequate level of F&V consumption, defined as per the recommendation guidelines for adult population. Participants were asked on how many days a week they consume F&V, and how many servings they consume on the days they include F&V in the meal. The World Health Organization (WHO) and Food and Agriculture Organisation (FAO) recommend consuming at least 5 servings of fruits and vegetables per day. Daily intake was calculated from these two questions and then categorised as: Adequate (minimum 5 servings/day) and Inadequate (<5 servings/day).

In order to measure the association with F&V with relevant sociodemographic factors, the following variables were included in the study based on their potential role in people’s dietary behaviour: Age groups (15-19/ 20-24/ 25-29/ 30-34/ 35-39/ 40-44/ >44); Residency (Urban/ Rural); Sex (male/ female); Marital status (never in union/married/in union/ divorced/other); Education (no education/ primary/ secondary/ higher); Religion (Christian/ Elcin/ other); Employment (Unemployed/ professional-technical-managerial agriculture/ other); Wealth status* (Poor-Non-poor).

*Wealth status is measure in all DHS surveys based on household ownership of durable goods, which are taken into account for calculating wealth scores by principle component analysis. The scores are then categorised into quintiles, with higher quintiles representing better wealth status. For this study wealth quintiles were merged into two categories: Q1+Q2 = Poor, Q3+Q4+Q5 = Non-poor.

### Data analysis

Data were analysed with IBM SPSS Statistics for Windows version 24.0 (IBM Inc., Armonk, NY, USA). Before analysis, the datasets merged (for men and women) and cleaned for missing values and outliers. Variables that were found to have significant collinearity were not included in the analysis. In order to account for the cluster sampling design, sampling strata and weight by using complex survey mode. The prevalence of adequate F&V consumption along with the control variables were described by percentages with 95% CIs. Regional differences in F&V consumption were calculated by cross-tabulation and were presented as boxplots and bar charts. To find the variables that independently predicted the adequate consumption of F&V, we performed a series of binary logistic regressions, first for the sample entire population and then for separately for men and women. Results were presented as Odds ratios and 95% CIs. All tests were two-tailed and was considered significant at alpha value of 5%.

## Results

### Descriptive statistics

In total 14 185 men and women were included in the study. Mean age for men and women was respectively 29.83 (SD 10.80) and 31.37 (SD 11.95) years.

Basis sociodemographic characteristics of the sample population were presented in Table 1. The prevalence of having ≥5 servings of F&V a day were respectively 4.2% among men (95% CI = 3.6-4.8) and 4.8% (95% CI = 3.7-6.2) among women. Those who had adequate amount of F&V were more likely to be in the age between 20-30 years, urban residents, had secondary level education, engaged in managerial/technical works, and living in the non-poor households.

**Figure 1:**
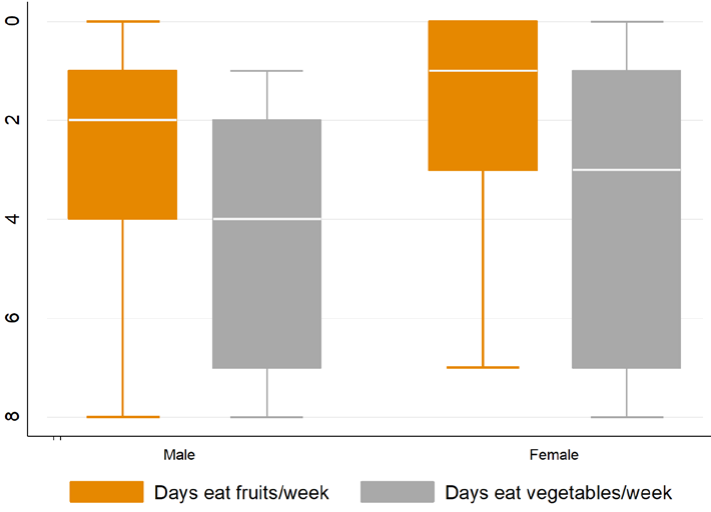
Prevalence of weekly F&V consumption among men and women in Namibia.

Figure 1 indicates the median number of days of eating fruits among men and women were respectively 2 and 4 days, and that of eating vegetables were respectively 3 and <2 days a week.

**Figure 2:**
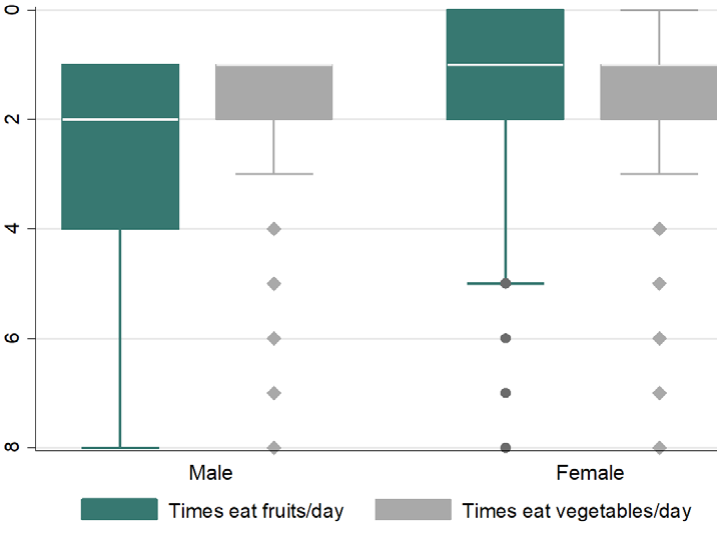
Prevalence of daily F&V consumption among men and women in Namibia.

As shown in Figure 2, the median number of times of eating fruits was higher among women, while that of eating vegetables was almost the same (<2 times/day).

**Figure 3:**
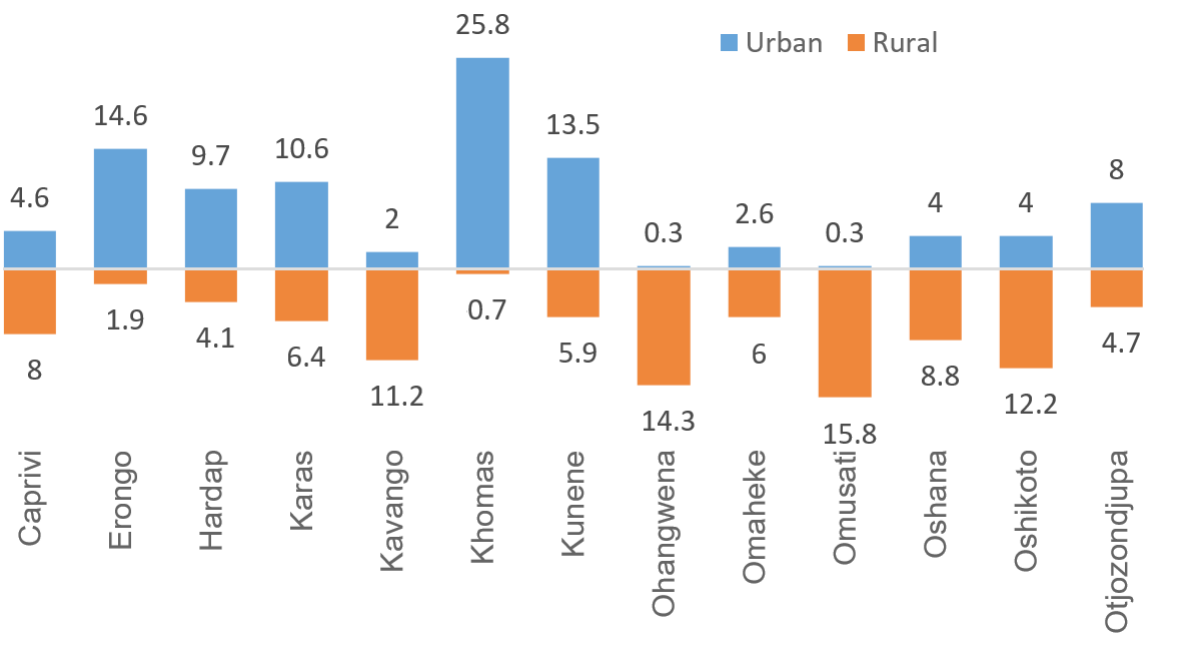
Regional difference in the prevalence of eating at least five servings of F&V in Namibia.

Figure 3 illustrates the regional variation in the prevalence of eating adequate amount of F&V per day. The highest prevalence was in the urban areas of Khomas (25.8%), and lowest in the urban areas of Omusati (0.3%). Overall, the highest prevalence was observed in Khomas (26.5%) and lowest in Omaheke (8.6%).

*Multivariable regression analysis*

The results of regression analysis on the association between F&V consumption and sociodemographic factors were presented in Table 2. Men and in rural areas had lower odds of eating at least 5 servings of F&V a day, however these odds were not statistically significant. Regarding educational level, the overall odds of eating at least 5 servings of F&V a day were higher among those who had primary level education (compared with those who had no education), with no significance observed after stratifying the results by sex. Of all the variables, the strongest association was found for wealth status, both among men and women. Overall, compared with participants who lived in the poor households, those who lived in the non-poor households were 2.3 times (1.59-3.37) as likely to eat at least 5 servings of F&V a day. After stratifying by sex, the corresponding figures for men and women were respectively 2.13 times (odds ratio [OR] =2.12, 95% CI=1.01-4.47) and 2.2 times (OR=2.19, 95% CI=1.56-3.38).

## Discussion

In this study we used a nationally representative survey to explore the prevalence of fruit and vegetable consumption among adult men and women in Namibia. Our findings indicated a remarkably low rate of F&V consumption among both men and women. Less than about 5% of the sample population consumed the combined amount of at least 5 servings of F&V per day as recommended by the WHO. In light of the present analysis it is hard to deduce the underlying causes behind the extremely low level of F&V consumption among Namibian adult population. Scarcity of studies from neighbouring countries also makes it difficult to show contrasting pictures. However, similarly low levels of F&V consumption were reported in Ghana (Demographic and Health Survey, 2013)^22^ and Mozambique (WHO STEPwise survey 2005).^23^ In Mozambique, less than 5% of the population were reported consume at least five servings of F&V per day, which is similar to the present findings for Namibian population. Another large-scale cross-sectional study in Ghana found that the mean number of F&V consumption per week was less than ten with significant differences among men and women. In both of these studies women were reported to have a higher likelihood of consuming at least 5 servings of F&V a day, whereas in the present study no such significant variation was observed. However, the percentage was slightly higher among women compared with men.

The prevalence was not only low for the overall population, but also varied significantly across the regions, and between urban and rural areas. For instance, the highest prevalence of consuming 5 servings of FNV was observed in the urban areas of Khomas (25.8%), and lowest in the urban areas of Omusati (0.3%). Owing to lack of data on total dietary composition it is not possible to estimate whether the regions with lower levels of F&V consumption had higher level of consuming animal-based food. One possible explanation could be persisting poverty, drought and food insecurity. Although the country has been one of the fastest growing economy in the continent, a considerable proportion of the population is still stuck in abject poverty (~28%) and malnutrition. Moreover, the food system is heavily reliant on food import which adversely affects local farming community in terms of livelihood and food security. In the multivariable analysis household poor wealth status was found to be strongly associated with lower consumption of F&V. Another factor might be the rising income levels among the wealthy communities which has been shown to be associated with increasing consumption of meat and dairy products.^24-26^ However, this aspect of low F&V consumption behavior is beyond the scope of the present study and needs to be explored by future studies.

The findings of the present study bear important messages for food and health policy making authorities in Namibia. While the country is still undergoing the HIV epidemic, chronic NCDs are also on the rise^27,28^ and is becoming a substantial contributor to morbidity and mortality burden in the population. This pattern is not unique in Namibia and has been reported in a large number of African countries.^29-33^ Low consumption of F&V is also being reported across the continent^34,35^ which is a growing concern for health as well as the agricultural sector. Poor dietary behavior, including inadequate F&V consumption is one the major risk factors for NCDs, hence needs to be given special policy attention. More studies and funding allocation should aim to explore the causes of low F&V intake and thereby adopt appropriate policy measure to address the situation.

As far we are concerned, this is the first population-based study exploring the prevalence and determinants of fruit and vegetable consumption in Namibia. It is also one of the few studies conducted on sub-Saharan African setting in this discipline. Sample size was considerably large and included both men and women. Data are also of high quality as DHS operates in about 90 countries and uses and standardised questionnaire for all surveys. We used carefully selected statistical methods for analysis to ensure a rigorous and comprehensive representation of the data. Nonetheless, there are several limiting factors inherent to source and type of the data that needs to be mentioned. Firstly, it is important to note that the survey was not particularly dedicated to collecting dietary behavior related information of the participants. This was the first DHS survey in Namibia that included a special section on fruit and vegetable consumption pattern among adult population. Therefore, it is understandable that analyzing the determinants of F&V consumption out of this dataset suffered the lack of more in-depth and precise information such as the types of fruits and vegetables, cooked or raw, processed or fresh, whether or not consumptions of juices were included which could have altered the prevalence to some extent. Moreover, people’s dietary behavior is determined greatly by local food production and procurement system. Although there were no data on such aspects, we have shown the regional differences in F&V consumption which can assist future studies investigating the geographical factors associated with availability and affordability of fresh F&V at community and national level. As the survey is few years old, the prevalence rates of F&V consumption might have changed and not reflect the current scenario. The data was cross-sectional and cannot reflect any causal relationship in the regression analysis.

## Conclusion

In conclusion, the findings of the study suggest that only a small proportion of the adult population is meeting the WHO recommended amount of daily F&V intake in Namibia. Significant disparities exist in the prevalence of regular F&V consumption as well, with urban and rural regions Caprivi, Kavango, Omaheke being characterised with the lowest rates. In this study we have included only F&V consumption as other dietary components were not available in the survey. Future studies should focus on total dietary intake and measure whether the low F&V intake is being compensated by higher amount of grain and animal-based food. Nutrition policy makes need to take urgent steps to inquire into the underlying causes and take strategic measures to promote F&V consumption with the goal of promoting general health and control rising prevalence of NCDs.

## Ethical approval

Ethical approval was not necessary for this study as the data were secondary and are available in public domain in anonymised form.

## Competing interests

The authors declare that they have no competing interests.

## Authors’ contributions

SY was responsible for collecting the dataset. SY and GB contributed to data analysis, interpretation and writing the manuscript text.

## Acknowledgments

We express sincere thanks to DHS for providing the datasets that made this study possible.

**Table 1:** Bivariate analysis between F&V consumption and the sociodemographic characteristics of the sample population (n = 14 185)

	**No., %**	**F&V intake**		***P***
		**<5/day** **95.7% (95.1-96.2)**	**≥5/day** **4.3% (3.8-4.9)**	
Age groups				0.251
15-19	2740, 19.3	21.6 (20.6-22.7)	16.6 (13.1-20.9)	
20-24	2491, 17.6	19.6 (18.6-20.5)	20.8 (16.5-25.8)	
25-29	2108, 14.9	16.0 (15.2-16.8)	14.2 (10.9-18.2)	
30-34	1778, 12.5	13.2 (12.4-14.1)	15.9 (12.0-20.8)	
35-39	1600, 11.3	11.7 (10.9-12.5)	9.7 (7.3-12.9)	
40-44	1346, 9.5	9.4 (8.8-10.2)	10.4 (6.9-15.5)	
>44	2122, 15	8.4 (7.7-9.1)	12.4 (9.0-16.7)	
Residency				<0.001
Urban	7256, 51.2	59.8 (57.9-61.7)	74.5 (69.2-79.2)	
Rural	6929, 48.8	40.2 (38.3-42.1)	25.5 (20.8-30.8)	
Sex				0.094
Male	4167, 29.4	27.3 (26.1-28.5)	30.1 (24.2-36.8)	
Female	10018,70.6	72.7 (71.5-73.9)	69.9 (63.2-75.8)	
Marital status				0.251
Never in union	6054, 42.7	43.0 (41.6-44.5)	38.4 (32.9-44.2)	
Married/in union	6970, 49.1	48.9 (47.4-50.3)	50.2 (44.8-55.6)	
Divorced/other	1161, 8.2	8.1 (7.4-8.8)	11.4 (8.3-15.5)	
Education				<0.001
No education	1142, 8.1	4.4 (3.9-5.0)	2.2 (1.0-4.5)	
Primary	3356, 23.7	19.4 (18.2-20.6)	12.1 (8.9-16.4)	
Secondary	8631, 60.8	65.7 (64.2-67.1)	67.7 (61.7-73.2)	
Higher	1056, 7.4	10.5 (9.1-12.2)	18.0 (13.6-23.5)	
Religion				0.617
Christian	5923, 41.8	39.8 (37.9-41.7)	40.1 (34.5-46.1)	
Elcin	5793, 40.8	44.5 (42.4-46.7)	40.4 (34.5-46.6)	
Other	2469, 17.4	15.7 (14.3-17.1)	19.5 (14.5-25.6)	
Employment				<0.001
Unemployed	7142, 50.3	48.8 (47.3-50.4)	37.7 (32.3-43.4)	
Professional/technical/managerial	3746, 26.4	28.0 (26.8-29.3)	38.4 (33.5-43.4)	
Agriculture/other	3297, 23.2	23.1 (21.8-24.6)	23.9 (19.4-29.2)	
Wealth status				<0.001
Poor	4896, 34.5	19.6 (18.6-20.5)	12.5 (8.9-17.2)	
Non-poor	9289, 65.5	16.0 (15.2-16.8)	87.5 (82.8-91.1)	

Note: Numbers represent percentages with 95% CI shown in parenthesis.

**Table 2:** Association between F&V consumption and sociodemographic factors in Namibia

	**Total** **OR (95% CI)**	**Male** **OR (95% CI)**	**Female** **OR (95% CI)**
Residency (Urban)			
Rural	0.80(0.61-1.05)	0.57(0.32-1.01)	0.57(0.32-1.01)
Education (no education)			
Higher	2.03(0.93-4.41)	0.77(0.29-2.00)	0.77(0.29-2.00)
Primary	1.59(1.03-2.45)*	1.58(0.68-3.67)	1.58(0.68-3.67)
Secondary	1.28(0.91-1.80)	1.12(0.58-2.15)	1.12(0.58-2.15)
Employment (unemployed)			
Professional/technical/managerial	1.11(0.81-1.52)	1.61(0.95-2.73)	1.61(0.95-2.73)
Agriculture/other	0.80(0.59-1.08)	1.07(0.63-1.81)	1.07(0.63-1.81)
Wealth index (poor)			
Non-poor	2.32(1.59-3.37)*	2.12(1.01-4.47)*	2.19(1.56-3.38)*

Abbreviations: OR, odds ratio; CI, confidence interval. * indicates statistically significant results. Reference categories are shown in parenthesis. Variables included in the model: Residency, Education, Employment, Wealth index.
